# Oral Hygiene in Adolescence: A Questionnaire-Based Study

**DOI:** 10.3390/ijerph19127381

**Published:** 2022-06-16

**Authors:** Luca Sbricoli, Lia Bernardi, Fatima Ezeddine, Christian Bacci, Adolfo Di Fiore

**Affiliations:** Department of Neurosciences, Dental Clinic, University of Padua, Via Giustiniani 3, 35100 Padova, Italy; liabernardi97@gmail.com (L.B.); f.ezeddine97@gmail.com (F.E.); christian.bacci@unipd.it (C.B.); adolfo.difiore@unipd.it (A.D.F.)

**Keywords:** orthodontics, adolescence, prevention, oral hygiene

## Abstract

Oral health is fundamental to our well-being, especially in adolescence. The aim of this study is to investigate oral hygiene knowledge through a questionnaire in a sample of adolescents, paying particular attention to those wearing orthodontic braces. The study was designed as a descriptive report of a local survey. An anonymous questionnaire was distributed to individuals born between 2000 and 2005, both on paper and online. Among 213 adolescents answering the questionnaire, 206 went to the dentist at least once (most of them between 5 and 9 years old), and 144 experienced at least one session of professional oral hygiene. Approximately 83% of the sample brushed their teeth at least twice a day, while only 7% used dental floss daily. Only 54% of respondents wearing orthodontic braces were advised to undergo professional oral hygiene during their orthodontic treatment. Education on oral hygiene at home came only from their parents for 61% of the whole sample. Most respondents had their first visit to the dentist apparently too late. Flossing was rare, whether the adolescents wore orthodontic braces or not. In many cases, professional oral hygiene was not common during orthodontic treatment. Adolescents did not learn about oral hygiene from a dental specialist.

## 1. Introduction

During adolescence, we grow up both physically and psychologically [1]. From a biological standpoint, adolescence coincides with an increase in hormone levels that leads to complete sexual maturity, which becomes apparent with a physical remodeling of adipose tissue and muscle, growth of genitals and development of secondary sexual characteristics [2]. Along with physical development, adolescents experience personal growth deriving from social, cognitive and emotional experiences.

Like every other part of the body, the oral cavity is also affected by physical and psychological changes associated with this phase of development. Increasing sexual hormones during puberty have a marked effect on the composition of subgingival bacterial flora showing higher levels of Gram-negative bacteria compared to earlier or later stages of life [3,4]. One of the first bacteria to be identified as growing, particularly in this age group, is *Prevotella intermedia*, which relies on estrogen and progesterone as growth factors [5]. Another type of bacterium that uses these hormones as growth factors is called *Porphyromonas intermedius*. This chromogenic bacterium tends to leave a black stain on the surface of the teeth, causing considerable esthetic damage. This micro-organism has also been isolated in children, but to a much lesser degree [5].

Nutrition has an important role in an individual’s health at every stage of life, but even more so at developmental age. A diet with a high intake of simple sugars contributes to the onset and progression of tooth decay [6]: according to the World Health Organization’s guidelines, these sugars should make up less than 10% of the total energy intake [7]. Adolescents also often have an inadequate intake of vitamin C, which has an important role as a cofactor in the formation of hydroxyproline, which is essential to the production of collagen [8]. The oral cavity is directly influenced by an individual’s diet but also by any related health issues. Eating disorders, such as anorexia and bulimia, damage the oral cavity involving salivary glands, oral mucosa and teeth: the most typical sign is dental erosion due to frequent episodes of self-induced vomiting [8].

Bad habits, such as smoking, drinking alcohol and abuse of narcotics, are mostly tried for the first time during adolescence and damage the body with repercussions that also become evident in the oral cavity. Studies have shown that smoking represents one of the main risk factors for periodontal disease because tobacco smoke alters the oral mucosa’s inflammatory response. The use of narcotics can influence the oral cavity in various ways causing extrinsic staining of the teeth, a reduced flow of saliva and a lower pH, oral mucosa erosion and ulceration, loss of the sense of taste and a generalized inflammation of the tissues supporting the teeth (gingivitis and periodontitis) due to poor oral hygiene and a very poor diet [9]. Oral cancer is among the most severe consequences of smoking, use of alcohol and narcotics [9,10].

Oral health is strongly influenced by an individual’s daily oral hygiene routine at home. Unless dental plaque is removed daily, it facilitates the onset of diseases of the gums and teeth (gingivitis, periodontitis and caries) [11].

Malocclusions may require orthodontic treatment with braces, which can have a negative effect on oral health by facilitating the accumulation of bacterial biofilm and hindering oral hygiene procedures at home [12]. Therefore, it is fundamental to ensure that adolescents wearing braces are given adequate instructions about oral hygiene and kept motivated by dental specialists [13].

Previous studies were found to assess oral hygiene focusing on adolescents: knowledge about this topic and oral hygiene routines varied throughout the literature. Many factors were proposed to explain the observed differences, such as socioeconomic status, gender element and geographical area of origin [12,14,15,16,17,18,19].

In the North-East region of Italy, a lot of interest is focused on improving preventive measures of the public health system. To assess the oral hygiene knowledge of young adults in this region, a questionnaire study was planned and subjected to patients attending a public clinic. Since no studies were conducted on this topic in this specific geographical region, the results from the present work could help implement preventive measures.

The aim of this study is to investigate oral hygiene knowledge in a sample of adolescents through a questionnaire examining actions they take and tools they use in their daily oral hygiene routines. The main variable considered is the past or present use of orthodontic braces to see how this might influence adolescents’ oral hygiene practices.

## 2. Materials and Methods

All patients attending the Borgo Cavalli Clinic in Treviso and the Dental Clinic of Padova University were considered as potential participants for the study to select a convenience sample of adolescent patients representing the Veneto region located in North-East Italy. Participants who were born before January 2000 and after December 2005 were excluded.

The questionnaire was distributed in an anonymous format, and it was administered in two ways: a printed paper copy and online through Google Forms platform. The questionnaire began with a brief explanation of the study’s purposes and asked respondents to answer as sincerely as possible. Before the beginning of the study, each patient was asked to provide an informed online consent explaining the purpose of the research and given authorization to use the collected data. Complete confidentiality was guaranteed to all patients.

The questionnaire was divided into four parts: general information, oral hygiene at home, professional oral hygiene and education on oral hygiene (Table 1). The questionnaire was made of one open question and 18 multiple-choice questions: 15 with only one possible answer and 2 with the opportunity to choose more than one. Some questions were linked to others, i.e., a positive or negative answer to one question enabled or denied the opportunity to answer the next one. The questionnaire was subjected to a pilot group of 40 patients for its validation; modifications were made where necessary to resolve ambiguities.

Data are presented with descriptive statistics indicating the frequency distribution of the answers.

## 3. Results

**Figure 1:**
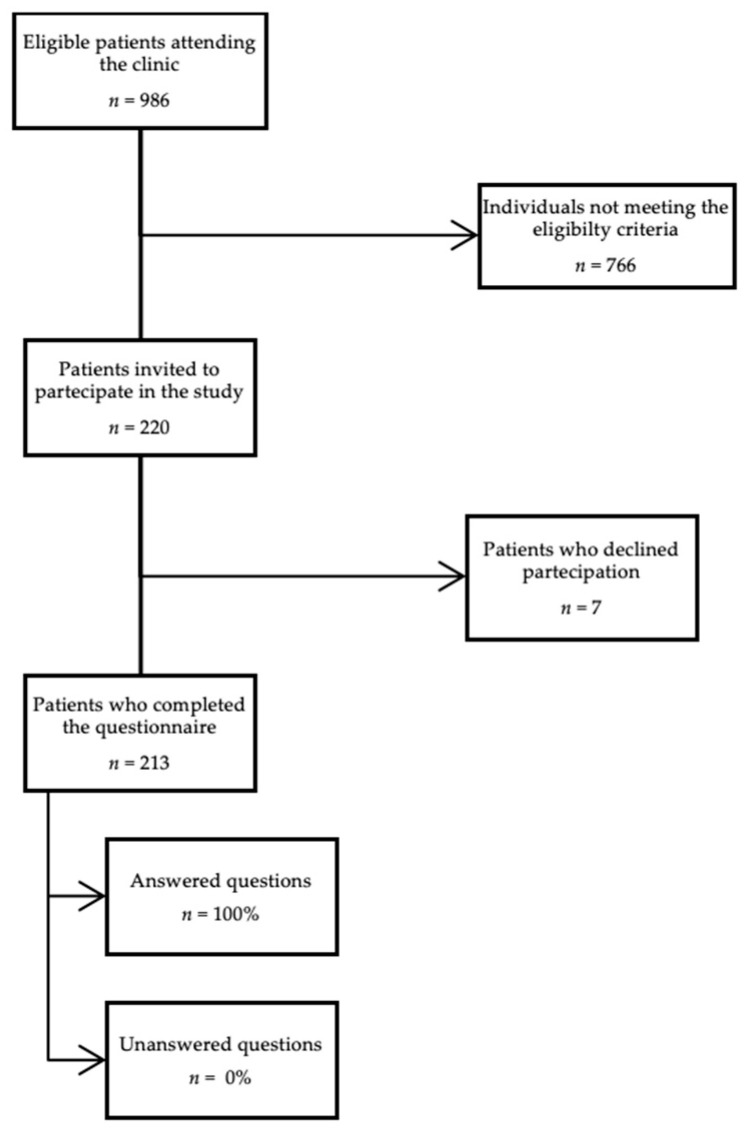
Flowchart of the study.

The questionnaire was completed by 213 adolescents; none of the questionnaires collected were rejected due to errors in their completion (Figure 1). The study population included 69 males (32%) and 144 females (68%); the mean age was 17 years old (range 14–19 years old).

**Figure 2:**
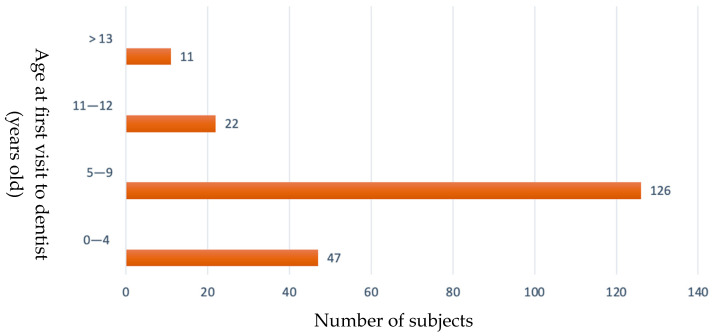
Age at first visit to dentist.

Of these 213 respondents, 206 (97%) reported having been to the dentist at least once in their life. When asked to specify at what age they first saw a dental specialist (choosing one of four age brackets), 61% (126 out of 206) declared that they were between 5 and 9 years old at the time (Figure 2).

### 3.1. Oral Hygiene at Home

Table 2 contains the information collected on respondents’ oral hygiene at home, distinguishing between males (69) and females (144) and between those who were wearing orthodontic braces (46) and those who were not (167).

The majority of respondents declared to use a manual toothbrush. A subset of adolescents used both manual and electric toothbrushes, while none of our respondents used a sonic toothbrush. More than 80% of the sample brushed their teeth at least twice a day, but only 7% used dental floss every day. Approximately half of the respondents reported routinely cleaning their tongues.

### 3.2. Professional Oral Hygiene

Differences regarding professional oral hygiene between adolescents with and without orthodontics are shown in Table 3. Overall, 83% of respondents knew about this procedure, and 67% experienced a session of professional oral hygiene with a dental hygienist at least once in their life. The frequency of these sessions varied considerably from one respondent to another. The study sample included 46 adolescents undergoing orthodontic treatment at the time and another 67 who had had orthodontic treatment in the past. Out of these 113 respondents, only 61 (54%) claimed to have been advised about submitting a session of professional oral hygiene during their orthodontic treatment (Figure 2).

### 3.3. Education on Oral Hygiene at Home

The last part of the questionnaire focused on education regarding appropriate oral hygiene practices. Respondents could choose among multiple answers. The collected data (see Table 4) show that most of the sample (128 adolescents) learned how to manage their oral hygiene exclusively from their parents and not from any other sources. There were 26 adolescents (12% of the sample) who reported having taken part in meetings on the topic of oral hygiene held outside the setting of the dentist’s surgery, and 175 (82%) indicated that they were interested in receiving more information on the topic.

## 4. Discussion

The present study aimed to investigate oral hygiene habits among adolescents in a regional contest in Italy through a questionnaire.

Results showed that almost all adolescents went to a dentist at least once in their lives (206 out of 213; 97%), and 126 out of 206 (61%) were between 5 and 9 years old at the time of their first visit.

Literature indicates that age at first dental visit widely ranges between 1 and 6 years worldwide. A recent questionnaire-based study conducted by Qu et al. (2022) [14] reported a mean age of 4.8 and 5.4 years old for preventive and symptomatic first dental visits, respectively. Furthermore, the study indicated that early preventive dental visits were associated with a lower rate of dental caries. Another study conducted by Alshahrani et al. (2018) [20] recorded a range of 3–6 years old at the time of the first dental visit. Mika et al. (2018) [21] reported a mean age of 3.79 years old at the time of the first dental visit, with most of the patients seeking dental treatment and only 36.9% for preventive measures. This suggests that the first dental visit occurs in a very wide age range, often in need of dental treatment, and there is no accordance between clinicians about the ideal age, regardless of country or region. Nevertheless, American guidelines suggest that the first dental visit should happen at the time of the first tooth eruption or no later than the first year of age.

Only 23% of our respondents met a dentist before they were five years old. There are various reasons to recommend regular visits to the dentist also for toddlers: (i) educate children and their parents to adopt good habits regarding their oral hygiene in order to maintain a good state of oral health in years to come, (ii) identify any lesions or disorders early on, and take corrective action as soon as possible, (iii) educate parents on the topic of nutrition and a balanced diet and (iv) establish a trusting relationship between the child and the dentist that will facilitate their trouble-free cooperation in the future [22]. Seeing a dentist for a check-up at a younger age also has a positive influence on the costs associated with dental treatments for children [23].

The present study produced some significant findings regarding adolescents’ oral hygiene at home in terms of the actions taken as part of their daily routine to look after their teeth and gums. According to the Italian National Guidelines for the promotion of oral health and the prevention of oral disease at developmental age, adolescents should brush their teeth twice a day. An ample majority (83%) of the adolescents in our sample reportedly complied with this recommendation, while the remainder (17%) only brushed their teeth in the morning or evening or not at all (only two adolescents reported never cleaning their teeth).

Brushing teeth is very important to remove plaque, but not enough. Some areas, such as the space between two adjacent teeth, are impossible to reach with a toothbrush, so other methods are required [24]. Adolescents should use dental floss daily; however, this study showed otherwise: only 35% of the subjects flossed often (only one subject flossed every day), while others did not like it (35%) or never tried it at all (30%).

According to Mottos-Silveira et al. [25], the main reason why flossing is so rarely performed lies in a lack of cooperation of the individuals, and any manual difficulty encountered with the flossing procedure itself would apparently be a minor issue. It is up to dentists and dental hygienists to educate adolescents and motivate them to make flossing a habit, also reminding the parents of its importance if necessary.

While results about brushing teeth in the present study mostly agreed with previous research, the frequency of interdental flossing varied throughout the literature.

Ericsson et al. (2012) [15] stated that 76% of adolescents brushed their teeth at least two times a day, with only 4% of the sample flossing every day. In a study conducted by Inquimbert et al. [19], the majority (69%) of adolescents declared brushing their teeth twice a day, with a higher frequency found in the subjects undergoing orthodontic treatment. Interdental flossing was still performed more often in the latter group compared to others (35.1% vs. 4.4%). A survey conducted among 879 adolescents from Portugal, Romania and Sweden reported that 77.4% brushed their teeth two or more times a day, while only 14.4% flossed daily (54% never used dental floss) [16]. Nasir et al. (2022) [17] stated that 66.8% of the sample reported brushing their teeth at least twice a day, while 14.2% reported at least once a day. In a study conducted by Veiga et al. (2014) [18] among Portuguese adolescents, results showed that 90.6% of the sample brushed their teeth twice a day or more, and 6% performed daily flossing.

When gender differences were considered, girls showed better compliance than boys in terms of both brushing their teeth and flossing (although the percentages for flossing were always very low). Girls were more attentive regarding tongue cleaning (an important step to effectively reduce bacterial load in the oral cavity and help prevent halitosis [26]).

Epidemiological studies point to a greater prevalence of gingivitis among males rather than females: one of the possible explanations lies in the amount of time spent on oral hygiene at home [27]. Other studies have also shown how girls usually have more knowledge and therefore pay more attention to oral hygiene procedures [15,28,29]. Our findings are consistent with the scientific literature, showing that adolescent females complied more with oral health care recommendations than their male counterparts, especially regarding cleaning interdental spaces and tongue.

The present study also found differences between adolescents with and without orthodontic braces: a better oral hygiene routine at home in the former can probably be explained by more frequent visits to the dental surgery. That said, the proportion of adolescents routinely using dental floss was still very low: only 10% of girls wearing braces were flossing regularly. Greater attention to oral hygiene procedures was found among adolescents that undergo orthodontic treatments compared to their counterparts [19].

Another finding that emerged from the questionnaire concerns the knowledge of what professional oral hygiene involves and the frequency of its sessions: 79% of our respondents reported knowing what it is, and 67% experienced a session of professional oral hygiene at least once in their life. However, the relationship between professional oral hygiene and orthodontic treatments seems to be particularly interesting: this supportive treatment was not provided for 46% of the adolescents wearing braces. This is particularly important while orthodontic treatments are underway, given the greater difficulty of daily teeth cleaning [30].

Providing instructions on how to manage oral hygiene at home is one of the main responsibilities of dentists and dental hygienists and a fundamental part of primary prevention strategies. When our sample of adolescents was asked to indicate who had taught them how to clean their teeth, 60% (128 respondents) reported having received instructions only from their parents. This finding is significant, keeping in mind that 206 out of 213 respondents went to a dentist at least once, and 143 attended a session of professional oral hygiene. This points to a failure on the part of dental professionals to educate their patients about oral hygiene. Instructing and motivating patients to routinely take care of their teeth and gums is a fundamental part of the dentist’s job and even more for dental hygienists. It is a fundamental primary prevention measure. Proper instructions enable individuals to maintain a healthy oral cavity, especially if they have received them already in childhood or adolescence. Our study findings also suggest that adolescents attribute importance to their oral hygiene, as 75% of our respondents reported being interested in having more information on the topic.

In this study, some limitations must be considered. First, a wider sample is needed to allow for generalization of the results; furthermore, the questionnaire was administered at a public dental hospital, limiting the population. Another limit is intrinsic to a research questionnaire-method based: self-reported data examined about childhood events (e.g., first visit to the dentist), making obtained data not completely reliable. Furthermore, clinical assessment of oral hygiene (e.g., bleeding on probing, plaque index) could have been helpful in comparing the data obtained from the questionnaire.

Future studies should be performed to investigate wider samples through validated questionnaires, possibly involving multiple clinical centers to minimize the geographic factor. Oral health indexes should be collected from clinical practice to compare them with the ones obtained through questionnaires.

## 5. Conclusions

Despite the limited number of patients interviewed, the present study produced some significant findings on oral hygiene behavior both at home and at the dental office. Most respondents claimed that they had their first visit to the dentist when aged between 5 and 9 years, which, according to the literature, seems to be too late. The majority of teenagers state toothbrushing as their only oral hygiene habit and therefore do not use any other oral hygiene item at home. Only a small percentage floss daily. Even adolescents wearing braces resulted in having inadequate compliance despite the closer recalls they need to undergo due to the ongoing orthodontic treatment. Professional oral hygiene is claimed to be widely known by the respondents; nonetheless, a significant amount of them never experienced it. Although orthodontic patients need to undergo this session even with a higher frequency compared to other patients, many dentists still do not suggest it during orthodontic treatments. Poor oral hygiene knowledge could increase the risk of dental caries in adulthood, as suggested by previous reviews. These findings should encourage the public health system to improve the preventive measure through better communication. Especially patients wearing braces should undergo a careful professional hygiene maintenance program to prevent tooth decay along with the orthodontic treatment.

## Figures and Tables

**Table 1:** Complete questionnaire subjected to all patients.

Questionnaire		
General information	Sex	(a) Male (b) Female
	Have you ever visited a dentist?	(a) Yes, I have (b) No, I have not
	If so, when was the first time you did it? (years old)	(a) Between 0 and 4 (b) Between 5 and 9 (c) Between 10 and 12 (d) Over 13
Oral hygiene at home	How many times a day do you brush your teeth?	(a) Never (b) Once (in the morning) (c) Once (in the evening) (d) Twice (both in the morning and in the evening) (e) Three times (f) More than three times
	What type of toothbrush do you use? (It is possible to choose more than one answer)	(a) Electric (b) Manual (c) Sonic
	Have you ever used dental floss?	(a) No, I have never used it (b) No, I tried it, but I did not like it (c) Yes, once a week (d) Yes, more than once a week (e) Yes, once a day (f) Other
	Do you use any other tools for your oral hygiene at home?	(a) Yes, I do (b) No, I do not
	If so, which ones?	…...............
	Do you also clean your tongue after tooth brushing?	(a) Yes, I do (b) No, I do not
Professional Oral Hygiene	Do you know what professional oral hygiene is?	(a) Yes, I do (b) No, I do not
	Have you ever undergone professional oral hygiene?	(a) Yes, I have (b) No, I have not
	If so, how many times a year?	(a) I did it only once (b) Less than once a year (c) Once a year (d) Twice a year (e) More than twice a year
	Do you wear braces?	(a) Yes, I do (b) No, I do not
	Have you ever worn it?	(a) Yes, I have. (b) No, I have not
	During the orthodontic treatment, has your dentist ever recommended that you undergo professional oral hygiene?	(a) Yes, he has (b) No, he has not
Oral hygiene instructions	Who provided you with training on oral hygiene? (It is possible to choose more than one option)	(a) My parents (b) No one (c) Dentist and/or oral hygienist (d) I found out information on the internet and on social networks
	Have you ever attended meetings on oral hygiene held by a dentist or dental hygiene at school or anywhere else?	(a) Yes, I have (b) No, I have not
	Would you like to receive more information about oral health?	(a) Yes, I would (b) No, I would not

**Table 2:** Oral hygiene at home: numbers are displayed as total and percentage of subjects.

	Total		Males		Females		Orthodontics		No Orthodontics	
Type of toothbrush										
Electric	29	(13.6%)	13	(18.8%)	16	(11.1%)	5	(10.9%)	24	(14.4%)
Manual	161	(75.6%)	56	(81.2%)	105	(72.9%)	34	(73.9%)	127	(76.0%)
Sonic	0	(0.0%)	0	(0.0%)	0	(0.0%)	0	(0.0%)	0	(0.0%)
Electric and manual	23	(10.8%)	0	(0.0%)	23	(16.0%)	7	(15.2%)	0	(0.0%)
Teeth brushing										
Never	2	(0.9%)	2	(2.9%)	0	(0.0%)	0	(0.0%)	2	(1.2%)
Once a day (morning)	24	(11.3%)	7	(10.1%)	17	(11.8%)	3	(6.5%)	21	(12.6%)
Once a day (evening)	10	(4.7%)	5	(7.2%)	5	(3.5%)	3	(6.5%)	7	(4.2%)
Twice a day	128	(60.1%)	46	(66.7%)	82	(56.9%)	28	(60.9%)	100	(59.9%)
Three times a day	46	(21.6%)	9	(13.0%)	37	(25.7%)	11	(23.9%)	35	(21.0%)
More than 3 times a day	3	(1.4%)	0	(0.0%)	3	(2.1%)	1	(2.2%)	2	(1.2%)
Flossing										
Never tried it	64	(30.0%)	24	(34.8%)	40	(27.8%)	12	(26.1%)	52	(31.1%)
No, I tried but did not like it	75	(35.2%)	27	(39.1%)	48	(33.3%)	13	(28.3%)	62	(37.1%)
Once a week	27	(12.7%)	9	(13.0%)	18	(12.5%)	7	(15.2%)	20	(12.0%)
More than once a week	20	(9.4%)	4	(5.8%)	16	(11.1%)	7	(15.2%)	13	(7.8%)
Once a day	15	(7.0%)	3	(4.3%)	12	(8.3%)	5	(10.9%)	10	(6.0%)
Other	12	(5.6%)	2	(2.9%)	10	(6.9%)	5	(10.9%)	7	(4.2%)
Tongue cleaning										
Yes	104	(48.8%)	29	(42.0%)	75	(52.1%)	26	(56.5%)	78	(46.7%)
No	109	(51.2%)	40	(58.0%)	69	(47.9%)	20	(43.5%)	89	(53.3%)

**Table 3:** Data obtained about professional oral hygiene.

Professional Oral Hygiene						
	Total		Orthodontics		No orthodontics	
	213 Subjects		46 Subjects		167 Subjects	
Awareness of professional oral hygiene	176	83%	35	76%	131	78%
Use of professional oral hygiene	143	67%	34	74%	109	65%
Frequency of use of professional oral hygiene						
*Once*	52	36%	9	20%	43	26%
*Less than once a year*	16	8%	5	11%	11	7%
*Once a year*	37	17%	9	20%	28	17%
*Twice a year*	32	15%	6	13%	26	16%
*More than twice a year*	6	3%	5	11%	1	1%

**Table 4:** Training on oral hygiene.

Training on Oral Hygiene		
	Total	
	213 Subjects	
Who provides training on oral hygiene		
*Parents*	128	60.1%
*Dentist or dental hygienist*	18	8.5%
*Parents and dentist or dental hygienist*	49	23.0%
*Parents, dentist or dental hygienist, and internet*	1	0.5%
*No training, internet*	1	0.5%
*No training*	13	6.1%
Attendance at meetings on oral hygiene		
*Yes*	26	12.2%
*No*	187	87.8%
Interest in obtaining more information about oral hygiene		
*Yes*	175	82.2%
*No*	38	17.8%

## Data Availability

The data presented in this study are available on request from the corresponding author.
